# Thoracic Aortic Dilation: Implications for Physical Activity and Sport Participation

**DOI:** 10.3390/diagnostics12061392

**Published:** 2022-06-04

**Authors:** Emanuele Monda, Federica Verrillo, Marta Rubino, Giuseppe Palmiero, Adelaide Fusco, Annapaola Cirillo, Martina Caiazza, Natale Guarnaccia, Alfredo Mauriello, Michele Lioncino, Alessia Perna, Gaetano Diana, Antonello D’Andrea, Eduardo Bossone, Paolo Calabrò, Giuseppe Limongelli

**Affiliations:** 1 Inherited and Rare Cardiovascular Diseases Unit, Department of Traslational Medical Sciences, University of Campania “Luigi Vanvitelli”, Monaldi Hospital, 80131 Naples, Italy; emanuelemonda@me.com (E.M.); fedeverrillo@gmail.com (F.V.); rubinomarta@libero.it (M.R.); g.palmiero@hotmail.it (G.P.); adelaidefusco@hotmail.it (A.F.); cirilloannapaola@gmail.com (A.C.); martina.caiazza@yahoo.it (M.C.); natale.0196@gmail.com (N.G.); alfredo.mauriello93@libero.it (A.M.); michelelioncino@icloud.com (M.L.); alessiaperna@hotmail.it (A.P.); gaetanodiana1991@gmail.com (G.D.); paolo.calabro@unicampania.it (P.C.); 2 Unit of Cardiology and Intensive Coronary Care, “Umberto I” Hospital, 84014 Nocera Inferiore, Italy; antonellodandrea@libero.it; 3 Cardiac Rehabilitation Unit, Cardarelli Hospital, 80131 Naples, Italy; ebossone@hotmail.com; 4 Institute of Cardiovascular Sciences, University College of London and St. Bartholomew’s Hospital, Grower Street, London WC1E 6DD, UK

**Keywords:** aortic disease, athletes, sport cardiology, bicuspid aortic valve, Marfan syndrome

## Abstract

Thoracic aortic dilatation is a progressive condition that results from aging and many pathological conditions (i.e., connective tissue, inflammatory, shear stress disorders, severe valvular heart disease) that induce degenerative changes in the elastic properties, leading to the loss of elasticity and compliance of the aortic wall. Mild aortic root enlargement may be also observed in athletes and is considered as a normal adaptation to regular exercise training. On the other hand, high-intensity physical activity in individuals with a particular genetic substrate, such as those carrying gene variants associated with Marfan syndrome or other inherited aortopathies, can favor an excessive aortic enlargement and trigger an acute aortic dissection. The evaluation of the aortic valve and aortic root diameters, as well as the detection of a disease-causing mutation for inherited aortic disease, should be followed by a tailored decision about sport eligibility. In addition, the risk of aortic complications associated with sport in patients with genetic aortic disease is poorly characterized and is often difficult to stratify for each individual athlete. This review aims to describe the relationship between regular physical activity and aortic dilation, focusing on patients with bicuspid aortic valve and inherited aortic disease, and discuss the implications in terms of aortic disease progression and sport participation.

## 1. Introduction

Athlete’s heart is a term used to define the complex of structural, functional, and electrical remodeling of the cardiovascular system induced by regular exercise training [1]. The type of cardiac remodeling depends on the type of physical exercise. Isotonic exercise is mainly responsible for a volume overload due to an increased cardiac output caused by an increased heart rate and stroke volume and reduced peripheral resistance with a moderate increase in systemic blood pressure. On the other hand, isometric exercise is associated with a pressure overload due to the significant increase in systolic and diastolic blood pressure, while no significant changes are found for heart rate, stroke volume, and cardiac output.

Most sports consist of a combination of isotonic and isometric muscular component exercises. The frequency, duration, and intensity of exercise are the main determinants of the cardiac remodeling observed in athletes. Thus, according to the type, frequency, duration, and intensity of muscular work maintained during the exercise program, sports are classified into four different categories (i.e., skill, power, mixed, and endurance) and three levels of intensity (i.e., low, medium, and high) [2].

The most well-known effects of regular physical activities on structural cardiac remodeling are those observed for the four cardiac chambers, especially in the left ventricle, with particular concern in terms of differential diagnosis with pathological conditions in extreme phenotypes [3,4,5]. Sports with a prevalent isotonic muscular component cause eccentric left ventricular hypertrophy, while those with a prevalent isometric muscular component are responsible for concentric left ventricular hypertrophy [1,6,7]. 

Another structural change which can be observed in athletes is the dilation of aortic root and ascending aorta [8].

Although evidence seems to suggest that aortic dilation in healthy athletes is generally a benign condition, physical activity in patients with a particular genetic substrate, such as gene variants associated with Marfan syndrome or other aortopathies, can favor an excessive aortic enlargement and trigger an acute aortic syndrome [9]. In particular, the increased systemic blood pressure and aortic stress during physical activity in patients with genetically triggered aortic disease could be responsible for aortic rupture or dissection, which is responsible for some cases of sudden cardiac death (SCD) in athletes [10]. Bicuspid aortic valve (BAV) represents another condition commonly associated with aortic dilatation and an increased risk of acute aortic syndromes [11]; thus, the identification of the aortic valve morphology is required in athletes participating in high-intensity sports.

The evaluation of the aortic valve and aortic root diameters, as well as the detection of a genetic basis linked to aortic disease, should be followed by a tailored decision about sport eligibility. Moreover, the risk associated with sport in patients with genetic aortic disease is well-known only for some inherited disorders, and it is often difficult to stratify for each individual athlete.

This review aims to describe the relationship between regular physical activity and aortic dilation, focusing on patients with BAV and genetic aortic diseases, and discuss the implications in terms of aortic disease progression and sport participation. 

## 2. Measuring the Aortic Root and Ascending Aorta

Aortic dilatation is a progressive condition that results from aging and many pathological conditions that induce degenerative changes in the elastic properties, leading to the loss of elasticity and compliance of the aortic wall [12]. Indeed, there is a linear relationship between maximal aortic diameter and aortic dissection or rupture risk [13].

Aortic size measurement is of paramount importance in diagnosing aortic dilatation, assessing the rate of increase over time, and identifying the cut-off points indicative of prophylactic intervention [14]. In adults, the aortic dimensions vary according to age and body size, and the upper limit of the average aortic diameter is defined as two standard deviations above the mean predicted diameter (Z score ≥ 2) [15]. However, there is poor agreement between different guidelines regarding average aortic size and the appropriate anatomical landmarks [16]. Recently, a height-based ratio (excluding weight and BSA calculations) yielded satisfactory results for evaluating the risk of natural complications in patients with thoracic ascending aortic aneurysm [17].

**Figure 1:**
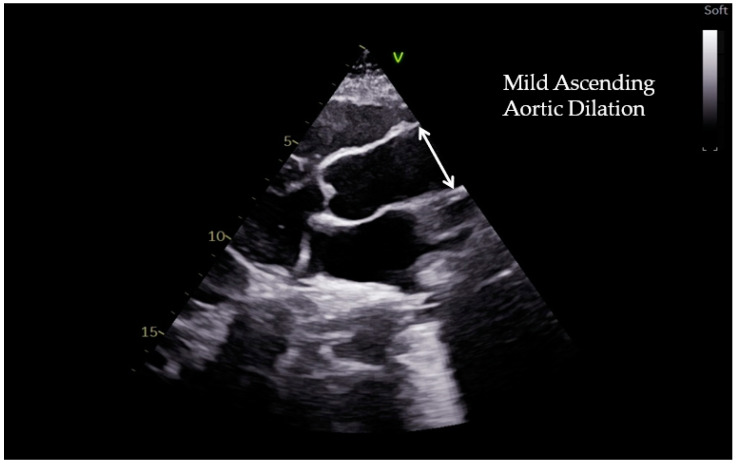
Long-axis view of echocardiography illustrating a case of mild ascending aortic dilation in a 11-year-old child with bicuspid aortic valve.

Following the last European guideline on diagnosing and managing aortic disease [9], echocardiography became the first imaging modality for screening aortic disease because of its availability, portability, and the possibility of evaluating other aspects, such as ventricular function and valvular disease. The reference values for measuring the aortic root and ascending aorta have been obtained from the two-dimensional (2D) parasternal long-axis view at pre-specified anatomical landmarks (aortic annulus, sinuses of Valsalva, sino-tubular junction, proximal ascending aorta) (Figure 1). 

**Figure 2:**
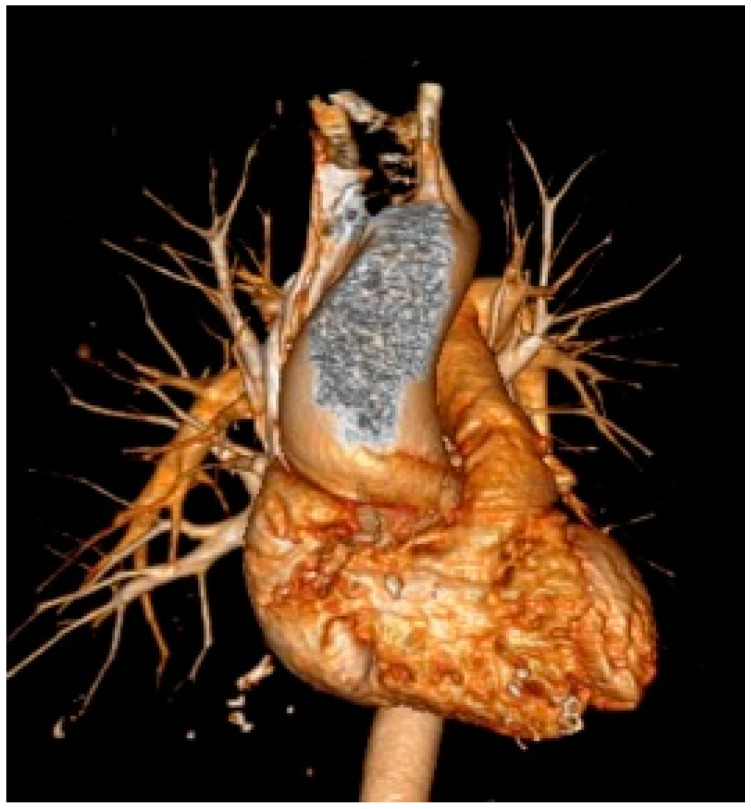
CT illustrating a case of ascending aortic aneurysm in a 65-year-old woman with bicuspid aortic valve.

This anterior-to-posterior aortic wall measurement in the end-diastole, perpendicular to the aorta longitudinal axis, is obtainable using a leading-edge-to-leading-edge method (as suggested for M-mode) or an inner-diameter-to-inner-diameter method [18], as proposed from some centers aiming to establish uniform TTE measurements with other imaging modalities. However, a single 2D view may underestimate the maximum annular dimension, especially if there is an asymmetric aortic enlargement. Moreover, as it is not a straight tube, the aorta may be imaged obliquely, overestimating its true diameter [19]. Finally, many other factors may limit the quality of the windows, ultimately limiting the accuracy of aortic measurement. Transesophageal echocardiography (TOE), computed tomography (CT), and magnetic resonance imaging (MRI) may overcome these limitations thanks to their higher spatial resolution and the possibility of performing a comprehensive multiplanar analysis using a three-dimensional (3D) dataset [20]. For both CT and MRI techniques, ECG-gated acquisition is advisable for improving the accuracy and reproducibility of the aortic measurements because cardiac pulsation and aortic motion may overestimate the maximum diameter. ECG-gated CT, given its higher spatial resolution, availability, and user independency, is the most accurate method for evaluating the aorta (Figure 2).

However, although single events present a low risk, radiation exposure could be harmful in younger patients requiring serial evaluations. This may be particularly important for women in whom frequent radiation exposure may increase risk of breast cancer. In these cases, and in cases in which contrast administration is contraindicated, MRI should be considered the first-line test [9]. 

A baseline evaluation with a CT scan or MRI is generally recommended in every patient with any sign of aortic disease, and a value of 40 mm at echocardiography should trigger the first evaluation. Furthermore, the detection of aortic dilatation requires a program of surveillance based on repetitive aortic measurements. 

Any technique can be helpful: echocardiography showed excellent accuracy and reproducibility in measuring aortic roots, while surveillance CT and MRI seem more robust in following ascending aorta dilatations. Therefore, for repetitive measures, we recommended using the imaging modality with the least amount of iatrogenic risk and, to overcome the differences existing in reporting and assessing measurements, use the same imaging modality [21]. Finally, aortic diameters could be indexed to body surface area, especially for the outliers in body size. However, there is still a lack of consensus regarding measurements used and whether those should be adjusted to body surface area (BSA), sex, and age [22].

Particular care should be given to postoperative aortic disease imaging evaluation. Since many different surgical techniques can be used for aortic repair, it is important to develop a dedicated expertise for normal postoperative morphologic findings and pathologic conditions in order to promptly identify patients at risk.

## 3. Aortic Dilation in Athletes

The aortic root and ascending aorta may respond to high levels of physical exercise, causing an enlargement of their diameters, but are rarely responsible for a dilation above the common threshold used for distinguishing pathology [8]. Thus, mild aortic root enlargement should be considered as a normal adaptation to regular exercise training.

Data on aortic diameters in athletes come from a small number of studies and one meta-analysis. Iskandar et al. performed a meta-analysis to evaluate the prevalence of increased aortic root dimension in athletes as compared with non-athlete controls [8]. They found a small (+3.2 mm (0.5 mm to 5.9 mm)) but statistically significant increase in the average aortic diameter in athletes. Moreover, they observed that a large increase in aortic diameters was unusual in athletes. 

In a large study evaluating 2317 highly trained competitive athletes, significant aortic root dilation, defined as ≥ 40 mm in males and ≥ 34 mm in females, was observed in 1.3% and 0.9% of the overall cohort, respectively [23]. Similarly, Gati et al. [24] reported a prevalence of aortic dilation in athletes of about 0.3%. Moreover, they observed that no significant changes in aortic diameter occurred among the athletes with aortic dilation over a 5-year follow-up period. 

Of clinical interest, no athletes described in the two previously mentioned studies showed an aortic diameter of more than 44 mm. This observation has a significant implication in terms of sport participation of athletes with mild aortic dilation (40–44 mm), with the possibility of reassuring athletes and clinicians about the benign clinical course of exercise-induced aortic dilation in healthy individuals, recommending period assessments.

The precise mechanism of the aortic enlargement in athletes is not fully understood. According to the data present in the current literature, the most important determinant of aortic dilation is considered the systemic blood pressure response to exercise. However, the combination of various other factors, such as the type of sport, intensity, duration, and genetic factors, plays an important role in determining aortic enlargement. A different study found gender, age, BSA, ethnicity, systemic blood pressure, left atrial diameter, left ventricular mass, and years of training as predictors of aortic dilation [23,24].

The type of sport also has a significant impact on the variability of aortic dimension, with endurance sports responsible for the largest impact on aortic root dimension [23,24]. In particular, the impact of sport on aortic dimensions has been recently addressed by Boraita et al. [25], who described the aortic root dimensions in elite athletes according to the type and intensity of the sport. They found that aortic dimensions were larger in athletes participating in sports with a high dynamic component. Similar observations were found by Pelliccia et al., who observed that male endurance athletes showed a larger aortic root diameter compared with male power athletes, while no significant differences were observed in female athletes [23]. Similarly, Gati et al. [24] found that male and female endurance athletes showed a trend towards a larger aortic diameter compared with those performing mixed or static sports.

According to the current guidelines, athletes can be classified as low, low-intermediate, intermediate, or high risk for acute aortic syndromes based on the valve morphology (i.e., tricuspid vs. bicuspid aortic valve), aortic diameter, and diagnosis of Marfan syndrome or other hereditary thoracic aortic diseases [2]. Athletes with a tricuspid aortic valve and aortic diameter <40 mm are considered to be at low risk, with the possibility of participating in all types of sports, although endurance is preferred over power sports. Patients with a low-intermediate risk are those with mild aortic dilation (40–45 mm), in whom high- and very high intensity exercise should be avoided. Finally, in athletes with an aortic diameter of 45–50 mm (intermediate risk) or >50 mm (high risk), sport should be avoided. Consideration for sport eligibility in BAV athletes or in individuals with Marfan syndromes or other aortopathies are discussed below.

## 4. Bicuspid Aortic Valve

BAV is a frequent congenital cardiac condition in both the general population, being reported in 0.5–2% of adults and 0.8% of newborns [26,27], and among competitive athletes, with an estimated prevalence of 2.5% [28]. 

**Figure 3:**
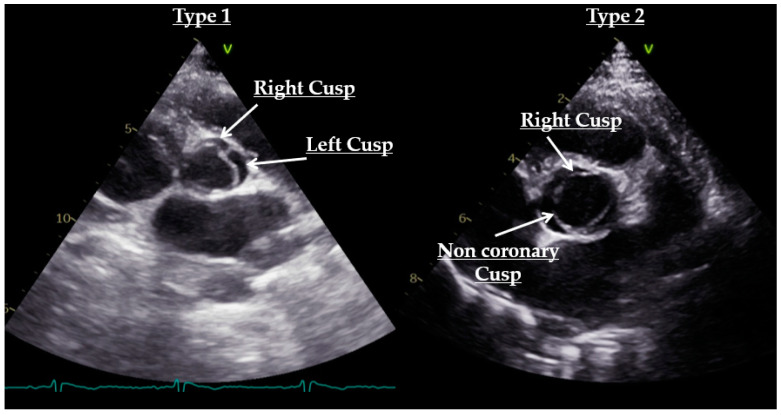
Short-axis view of echocardiography illustrating two cases of bicuspid aortic valve: type 1, with the fusion pattern involving the right and left cusps (on the **left**); type 2, with fusion pattern involving the right and non-coronary cusps (on the **right**).

**Figure 4:**
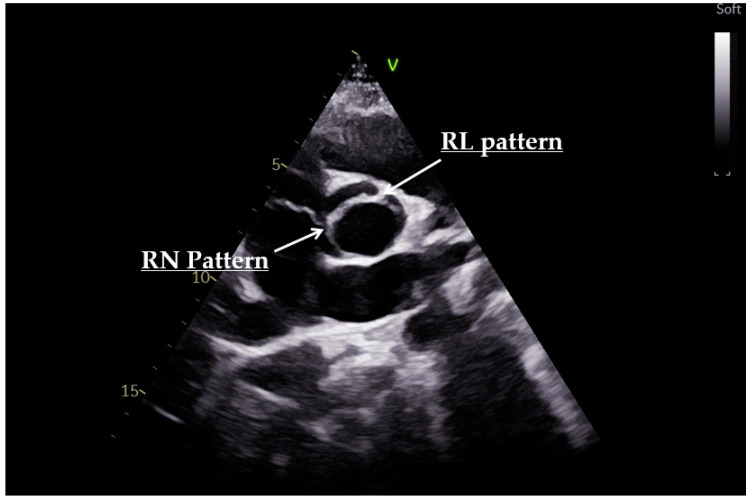
Short-axis view of echocardiography illustrating an aortic valve with presence of two raphes resulting in a restricted orifice area (right–left (RL) pattern and right–non-coronary (RN) pattern).

The classification of Sievers and Schmidtke based on raphes number and position is used to classify BAV into three phenotypes [29]. The most common fusion pattern, type 1, involving the right and left cusps, results in an anterior–posterior leaflet orientation. Type 2, involving the fusion of the right and non-coronary cusps, results in a right–left leaflet orientation. The less common type, type 3, is characterized by the fusion of the left and non-coronary cusps [27] (Figure 3). Although rare, it is possible to observe the presence of two raphes resulting in a restricted orifice area that extends from the periphery to the center (Figure 4).

BAV is recognized as a valvulo-aortopathy since aortic valve dysfunction (aortic stenosis (AS) or aortic regurgitation (AR)) and ascending aorta dilation are the most frequent associated complications [30]. The type and prevalence of aortic valve dysfunction or aortic dilation depend on the type of BAV. In particular, disease progression or complications may be of greater significance in patients with greater closure line eccentricity and an anteroposterior-oriented line of closure [31]. Furthermore, aortic dilation, which is present in nearly half of all patients with BAV, is considered a risk factor for aortic dissection/rupture (estimated to be present in up to 9% of BAV patients) [32,33] and SCD [34]. 

As genetic and hemodynamic components are implicated in progression of BAV valvulo-aortopathy [35], it is generally believed that intense physical activity may impair hemodynamic conditions, leading to aortic dilation and placing athletes at high risk of complications [36]. Nevertheless, sports-related SCD is very uncommon among athletes with valvulo-aortopathy, representing about 5% of cardiovascular causes of death [37,38,39]. Interestingly, aortic root measurements of elite athletes with a tricuspid aortic valve are within the normal values for the general population, suggesting that sport activity may not substantially alter aortic dimensions [35], as previously discussed.

Overall, AS is the most frequent complication, involving about 50% of patients with BAV, being more frequent in older individuals with fibrosis and cusp calcifications than individuals with tricuspid valves [40]. On the contrast, AR occurs more frequently in younger patients and is considered a proxy for endocarditis [41]. Prevalence of dilatation of the ascending aorta among patients with BAV can be as high as 80% [42]. The incidence of aortic dissection is low but still higher than in the general population (3.1 cases per 10,000 patients per year) [39].

Abnormal valve dynamics associated with BAV lead to aortopathy and even normally functioning BAVs can have abnormal transvalvular flow patterns, resulting in increased wall shear stress, which can be largely predicted by BAV morphology [43].

However, despite its frequency in the general population, the natural history of athletes diagnosed with BAV is limited, and it is not known whether restriction of physical activity limits the risk or the rate of aortic enlargement or dissection in either children or adults [44,45].

For instance, dimensions of aortic annulus, Valsalva sinuses, the sino-tubular junction and proximal ascending aorta measured longitudinally for 5 years in BAV individuals showed a progressive enlargement with no differences between athletes and sedentary subjects [46]. Only one study evaluated the association of long-term athletic training on the clinical course of BAV in a group of 81 Olympic athletes with BAV followed for a mean of 13 years [47]. Based on clinical and echocardiographic criteria, athletes were initially divided into low-risk (athletes deemed eligible for competitive sport after the first evaluation) and high-risk groups (presence of moderate-to-severe aortic stenosis/regurgitation, aortic ectasia, or signs of left ventricular remodeling). Moreover, the authors observed that, in high-risk athletes with BAV, the long-term progression of valvular disease occurred even after disqualification from competitive sports, while most low-risk athletes (88%) had an unremarkable clinical course.

Evidence suggests that exercise may not have a negative impact on left ventricle structure and function [46,48], although the length of follow-up and number of athletes followed longitudinally vary considerably. In a study evaluating 292 subjects with BAV (of whom 210 were athletes, 23 ex-athletes, and 59 healthy controls) no significant variation in left ventricular morphology and function was observed over 5 years [49]. Similar results were found in children diagnosed with BAV, where the prevalence and degree of aortic diameter progression were not significantly different between physically active and sedentary subjects over a 2-year period, suggesting that aortic dilation is unrelated to regular physical activity [50].

As such, access to competitive sports for individuals with BAV can be granted after a careful consideration of symptoms, functional capacity, type and severity of aortic valve disease, changes in myocardial structure, arrhythmias, and left ventricular remodeling and function. 

Recently, the European Society of Cardiology (ESC) issued new recommendations regarding sport eligibility of patients with BAV (summarized in Table 1) [2]. In general, in the absence of evident aortopathy, exercise recommendations for individuals with BAV are identical to those for individuals with tricuspid aortic valve dysfunction [2]. Symptomatic individuals with BAV presenting with mild AS or AR are eligible for all recreational/competitive sports, although regular follow-ups are still warranted. Eligibility is limited to moderate-to-low intensity sports or not recommended with valvulopathy worsening.

Although practicing sport may be safe for patients with the mild BAV phenotype, it is worth noting that in the longest follow-up study [47], up to one-in-eight individuals with low-risk BAV developed disease progression in a time window of 7–17 years from first clinical evaluation. As such, close monitoring of morphology, arrhythmic profile, and functional capacity is mandatory to intercept early but significant signs of disease progression. 

## 5. Heritable Thoracic Aortic Disease

Heritable thoracic aortic disease (HTAD) comprises a large spectrum of diseases defined by the occurrence of aortic disease, mainly aneurysm or dissection. HTAD can be classified as non-syndromic, when the disorder is limited to aortic disease, and syndromic, when extra-aortic features are included [51]. In patients with non-syndromic aortic disorder, about one-third exhibit a family history of aortic disease, which indicates a significant genetic component [52]. However, the genetic basis of non-syndromic disease is complex, and a pathogenic variant is detected in only up to 20% of patients [53]. On the other hand, a strong gene–disease association can be observed in syndromic aortic diseases, such Marfan syndrome.

Marfan syndrome is a rare autosomal dominant systemic disorder with a prevalence of 1–5 cases among 10,000 people. However, the prevalence could be higher in athletes participating in sports, such as volleyball, basketball, and high jump [54]. 

Up to 95% of patients with Marfan syndrome have a disease-causing mutation in FBN1, leading to a pathogenic alteration in the extracellular matrix protein fibrillin 1 [55]. Fibrillin microfibrils are extensible polymers with a structural role in the extracellular matrix that endow connective tissues with long-term elasticity. Moreover, they have a large distribution in connective tissue, both elastic and non-elastic. Microfibrils have an important role in bio-signaling (regulating the transforming growth factor beta (TGFβ)) and mechano-signaling (in assisting local response to hemodynamic changes) [56]. 

**Figure 5:**
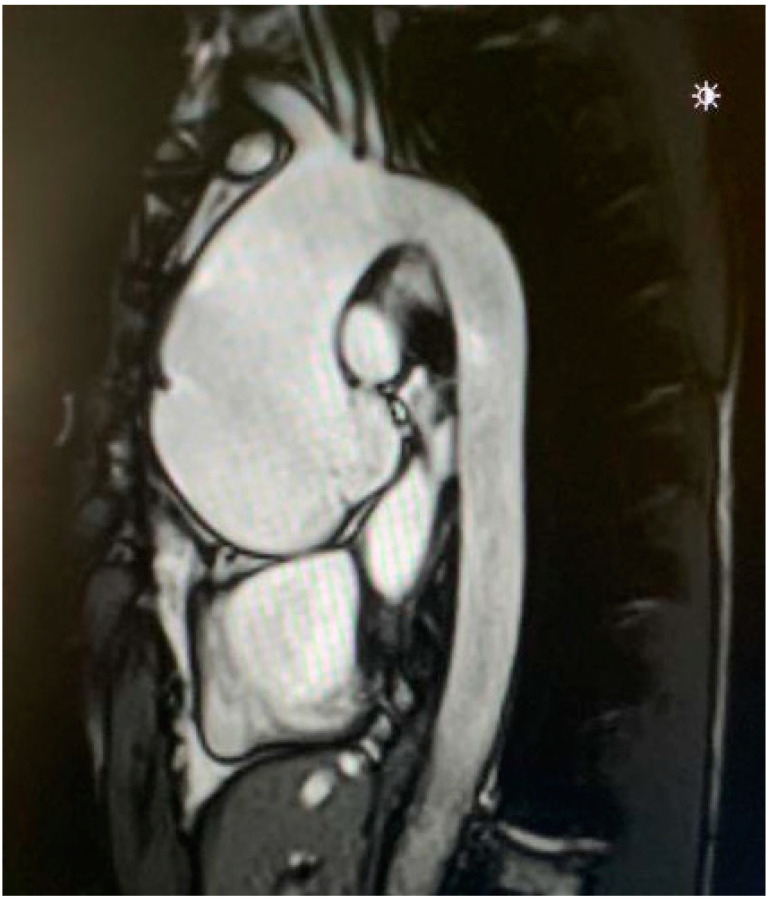
Cardiac magnetic resonance imaging showing a severe aortic root dilation in a patient with Marfan syndrome.

The main manifestations involve the cardiovascular system (including mitral regurgitation, caused by valve prolapse, and aortic root dilatation) and skeletal and eyes abnormalities. The aortopathy, responsible for progressive aortic dilation, leading, in severe cases, to aortic dissection, is the main cause of morbidity and mortality in patients with Marfan syndrome (Figure 5) [57]. Diagnosis of Marfan syndrome can be made by clinical manifestations, family history, and genetic analysis according to the Ghent criteria [57,58].

In the literature, there are few studies evaluating Marfan syndrome in the endurance athletic population. Three studies, of which only one was carried out in humans, have proposed training protocols with the purpose of observing the effect of physical activity on aortic function and structure. Gibson et al. [59] studied Marfan and wild-type mice, which were subjected to voluntary (cage wheel) and forced (treadmill) exercise regimens or a sedentary lifestyle for 5 months. They observed a biphasic effect of aerobic exercise on aortic structure and function, with an optimum protective effect at low-intensity exercise (55–65% VO2 max) and tapering off at high-intensity (85% VO2 max). These findings support the role of mild-to-moderate aerobic exercise in improving aortic health and reducing its susceptibly to rupture by reducing elastin fragmentation. Similarly, Mas-Stachurska et al. [60] evaluated the effect of moderate aerobic activity on the aortic growth in Marfan mice over a 5-month period. They noted that exercise significantly blunted the aortic root dilation rate in Marfan syndrome mice compared with sedentary mice, confirming the potential benefit of moderate aerobic activity in Marfan syndrome. Finally, Benninghoven et al. [61] carried out a 3-week rehabilitation protocol in 18 patients with Marfan syndrome who previously underwent cardiovascular surgery. Several benefits in terms of physical fitness, heart-related quality of life, and psychological wellbeing were observed. In conclusion, current data emerging from mice model studies show positive results in terms of aortic structure and function improvement. However, large cohort studies on humans are required to confirm these results.

In patients with Marfan syndrome, intense physical activity is generally discouraged due to the risk of the progression of aortic dilation and aortic rupture. Thus, the involvement of individuals with Marfan syndrome should be limited to low-intensity skill sports (e.g., golf, bowling) in order to avoid an excessive increase in systemic blood pressure, associated with endurance or power sports, which could trigger an acute aortic syndrome [2,9]. Moreover, individuals with Marfan syndrome should avoid contact sports such as boxing or football due to their skeletal and cardiovascular susceptibilities [62].

According to the current ESC guidelines [2], individuals with Marfan syndrome without aortic dilatation are classified at low-intermediate risk, and these individuals are recommended to avoid high- and very high intensity exercise, other than contact and power sports, with a preference for endurance sports. Table 2 summarizes current ESC recommendations regarding sports activity and surgery in patients with Marfan syndrome.

## 6. Future Direction

The genetic landscape of aortic diseases is rapidly increasing with expanding knowledge being clinically actionable. Most HTAD genes can be classified into three groups: extracellular matrix proteins, vascular smooth muscle cells, and TGF B signaling pathway. A consensus based on experts’ opinions is available for selecting patients in whom genetic testing should be performed: familiar thoracic aortic diseases, aortic dissection, or aortic root diameter Z score >_3 in childhood and aortic dissection or aortic root diameter Z score > 3.5 in adults [63]. To date, major goals of genetic testing are performing an effective family screening, given the extreme variability of phenotypes even among family members, and determining the optimal management for each patient.

Future research will be necessary to tailor genetic mutations to sports activity recommendations and identify those variants associated with a higher risk of aortic syndromes and emergencies. 

## 7. Conclusions

The aortic root and ascending aorta may respond to high levels of physical exercise, causing an enlargement of their diameters, occasionally, although rarely, resulting in a dilation above the common threshold used for distinguishing pathology from physiology. In healthy athletes, the identification of mild aortic dilation could be considered a normal adaptation to regular exercise training. On the other hand, in individuals with aortic dilation, a genetic disease should be excluded, since it could have significant implications in terms of aortic disease progression and sport participation.

## Figures and Tables

**Table 1:** Eligibility for sport participation of patients with bicuspid aortic valve in sport activity according to the 2020 ESC Guidelines on sports cardiology and exercise in patients with cardiovascular disease (2). Abbreviations: BP, blood pressure; LoE, level of evidence. LVEF, left ventricular ejection fraction. Class of recommendation: Class I, green color; Class IIa, yellow color; Class IIb, orange color; Class III, red color.

	Recreational Sport	Competitive Sport	
Aortic Stenosis			
Mild	All sports Class I; LoE C	All sports Class I; LoE C	
Moderate	Low-moderate intensity LVEF > 50%, good functional capacity, and normal exercise test. Class IIa; LoE C	Low-moderate intensity LVEF > 50%, good functional capacity, and normal exercise test. Class IIb; LoE C	
Severe	Low intensity LVEF > 50% and normal BP response during exercise. Class IIb; LoE C	Low intensity LVEF > 50% and normal BP response during exercise. Class IIb; LoE C	
	Moderate and high intensity is not recommended for individuals with LVEF < 50% and/or exercise-induced arrhythmias. Class III; LoE C	Moderate and high intensity is not recommended for individuals with LVEF < 50% and/or exercise-induced arrhythmias. Class III; LoE C	
Aortic Regurgitation			
Mild	All sports Class I; LoE C	All sports Class I; LoE C	
Moderate	All sports should be considered non-dilated LV with LVEF > 50% and normal exercise stress test. Class IIa; LoE C	All sports should be considered for individuals with LVEF > 50% and normal exercise test. Class IIa; LoE C	
Severe	Low and moderate intensity may be considered for individuals with a mild or moderately dilated LV with LVEF > 50% and normal exercise stress test. Class IIb; LoE C	Low and moderate intensity may be considered for individuals with a mild or moderately dilated LV with LVEF > 50% and normal exercise stress test. Class IIb; LoE C	
	Moderate or high-intensity is not recommended for individuals with LVEF < 50% and/or exercise-induced arrhythmias. Class III; LoE C	Moderate or high intensity is not recommended for individuals with severe AR and/or LVEF < 50% and/or exercise-induced arrhythmias. Class III; LoE C	
Aortopathy	Sport Category		
	Low Intensity	Intermediate Intensity	High Intensity
<40 mm LOW RISK	All sports are permitted with preference for endurance over power sports; follow-up every 2–3 y		
40–45 mm LOW-INTERMEDIATE RISK	Avoid high- and very high intensity exercise, contact, and power sports; endurance sports are preferred over power sports. Follow-up every 1–2 y		
45–50 mm INTERMEDIATE RISK	Only skill sports or mixed and endurance sports at low intensity are permitted. Follow-up every 6–12 months		
>50 mm HIGH RISK	Sports are (temporarily) contraindicated. Follow-up after treatment		

**Table 2:** Eligibility for sport participation and aortic surgery in patients with Marfan syndrome. Class of recommendation: Class I, green color; Class IIa, yellow color; Class IIb, orange color; Class III, red color.

Recommendations for Sports and Surgery in Marfan Syndrome				
	**<40 mm**	**40–45 mm**	**45–49 mm**	**≥50**
**Advice**	Avoid high- and very high intensity exercise, contact, and power sports. Preference for endurance over power sports	Only skill sports or mixed or endurance at low intensity	No sport recommended	
**Follow-up**	1–2 years	6 months–1 year	6 months	Re-evaluate after surgery
**Surgery**		≥45 surgery recommended if ≥1 high-risk factor. High-risk factors for Marfan syndrome patients are: (a) Aortic diameter at the sinuses of Valsalva ≥5 cm; (b) Rapid increase in aortic dilatation (≥3 mm per year); (c) Family history of aortic dissection at a low aortic size; (d) Progressive aortic regurgitation; (e) Personal history of spontaneous vascular dissection and (f) Desire for pregnancy.		Surgery is indicated
